# Protein alterations in women with chronic widespread pain – An explorative proteomic study of the trapezius muscle

**DOI:** 10.1038/srep11894

**Published:** 2015-07-07

**Authors:** Patrik Olausson, Björn Gerdle, Nazdar Ghafouri, Dick Sjöström, Emelie Blixt, Bijar Ghafouri

**Affiliations:** 1 Division of Community Medicine, Department of Medical and Health Sciences, Faculty of Health Sciences, Linköping University and Pain and Rehabilitation Center, Anaesthetics, Operations and Specialty Surgery Center, Region Östergötland.

## Abstract

Chronic widespread pain (CWP) has a high prevalence in the population and is associated with prominent negative individual and societal consequences. There is no clear consensus concerning the etiology behind CWP although alterations in the central processing of nociception maintained by peripheral nociceptive input has been suggested. Here, we use proteomics to study protein changes in trapezius muscle from 18 female patients diagnosed with CWP compared to 19 healthy female subjects. The 2-dimensional gel electrophoresis (2-DE) in combination with multivariate statistical analyses revealed 17 proteins to be differently expressed between the two groups. Proteins were identified by mass spectrometry. Many of the proteins are important enzymes in metabolic pathways like the glycolysis and gluconeogenesis. Other proteins are associated with muscle damage, muscle recovery, stress and inflammation. The altered expressed levels of these proteins suggest abnormalities and metabolic changes in the myalgic trapezius muscle in CWP. Taken together, this study gives further support that peripheral factors may be of importance in maintaining CWP.

The prevalence of chronic widespread pain (CWP) approximates up to and around 10% of the general population^1^,^2^,^3^,^4^ of which 10–20% are also diagnosed with fibromyalgia syndrome (FMS)^5^,^6^,^7^. The clinical pictures of CWP and FMS are dominated by the widespread pain but generally also include other somatic and psychological symptoms e.g. depressive symptoms, tiredness, unrefreshing sleep, reduced cognitive capacity. The risk factors for the transition from a local pain condition to CWP are largely unknown but according to a recent systematic review^8^ some studies claim increasing age, female gender, low physical activity, obesity and depression as risk factors^3^,^4^. There is a mutual agreement that CWP/FMS is characterized by signs of central hyperexcitability and alterations in the pain matrix in the brain^5^,^9^,^10^,^11^,^12^. Other possibly related characteristics are disturbances in neuroendocrine and autonomic nervous system^13^,^14^. There is a debate concerning whether peripheral factors are of importance or not for maintaining the central alterations. Hence, there are reports indicating peripheral alterations^15^,^16^,^17^, which may act as pain generators in CWP/FMS, such as mitochondrial disturbances in type-I muscle fibres, increased DNA fragmentation of muscle nuclei, alterations in the mitochondrial respiration chain, reduced capillarization and altered microcirculation, and greater thickness of the endothelium of the capillaries^18^,^19^,^20^,^21^,^22^,^23^. Recently three research groups have reported alterations of the peripheral nociceptors in FMS^16^,^24^,^25^. Various treatment studies also indicate an important role of peripheral input in maintaining pain and central alterations in CWP/FMS^26^,^27^,^28^. These studies give further support to the hypothesis that peripheral factors contribute to the maintenance of pain in CWP/FMS.

Human skeletal muscle is a heterogeneous tissue and is characterized by the capacity to adjust size and functionality due to both endogenous and exogenous influences. Substantial muscle proteomic studies have covered areas such as chronic^29^ and subacute hypoxia^30^, aging^31^,^32^,^33^ cold- and overfeeding^34^,^35^, exercise training^36^,^37^,^38^,^39^,^40^ and to a much lesser extent chronic pain^41^. In a recent study^42^ we have identified proteins from the interstitium of trapezius muscle in women with chronic regional myalgia or with CWP/FMS using microdialysis (MD), which is a method originally used studying metabolites^43^. Several of the identified proteins were at least 2-fold up or down-regulated in the chronic myalgia group compared to healthy controls. Furthermore, many of these proteins are known to be involved in nociceptive processes e.g. nerve growth factor (NGF), creatine kinase and fatty acid binding protein^42^. The neurotrophin NGF and other neurotransmitters such as substance P and calcitonin gene-related peptide (CGRP) are not only associated with the sensitivity of nociceptors but are also involved in inflammatory response^44^,^45^.

Two dimensional gel electrophoresis (2-DE)^46^ is widely used for separating and quantifying proteins from different tissues or fluids. Although in the later years mass spectrometry (MS) has developed and expanded the proteomic field, 2-DE is still the golden standard and a powerful separation method that can resolve complex mixtures of proteins. Although this impartial approach, of detecting a large scale of the proteome of any given tissue or body fluid can result in an overwhelming amount of data. Traditional statistical methods assume variable independence and disregard interrelationships between variables. As a growing belief that a panel of multiple biomarkers, or biocluster will perform better than a single biomarker in the attempt of understanding the pain mechanisms the development of large-scale-data analyses or multivariate data analyses (MVDA) has emerged. These statistical methods are capable of handling a number of intercorrelated substances and uses advanced principal component analyses (PCA) and Partial Least Squares (PLS) regressions as important tools. These methods can be applied on large data sets which can handle low subject-to-variables ratios. This complementary MVDA approach which also considers the internal relationship between variables, therefore reducing the multiple testing issues, which taken together creates a potential for a better understanding of the complex biochemical changes that may occur in chronic musculoskeletal pain.

Proteomic studies have the potential to significantly improve our knowledge of peripheral muscle alterations and its relation to aspects of pain in CWP and hopefully open up for mechanism-based classifications and treatments of common chronic pain conditions. Hence, this cross-sectional study investigates the proteome of trapezius muscle biopsies in women with CWP compared to pain-free female controls (CON) using 2-DE in combination with MVDA.

## Material and Methods

### Subjects

Primary screening was accomplished using a self-reported pain questionnaire and a structured telephone interview. To confirm the individual eligibility, all potential subjects were invited to answer a brief questionnaire concerning their health condition and to receive a standardized clinical examination that included weight, height, and blood pressure measurements. The questionnaire has recently been described^47^ and included questions concerning habitual pain intensity in the neck –shoulder region and low back using a numeric rating scale (NRS) (0 = no pain and 10 = worst possible pain) for neck, shoulders, and low back over the previous seven days. The questionnaire also included items concerning psychological aspects (anxiety, depression and catastrophizing) and quality of life; for details about these instruments see^47^. For both groups of potential subjects, a clinical examination was used to check inclusion and exclusion criteria (see below). The American College of Rheumatology (ACR) criteria were used for definition of CWP and/or FMS^48^. All subjects were clinically examined by either of two physicians.

Patients with CWP were recruited for the study either among former patients with CWP at the Pain and Rehabilitation Centre of the University Hospital, Linköping, Sweden or from an organization for FMS patients. As a result of this recruitment process, 18 women with CWP agreed to participate in the study. Inclusion criteria were female sex, age between 20 and 65 years, and widespread pain according to the ACR criteria. 15 out of these also fulfilled the criteria for FMS.

The 19 healthy controls (CON) were recruited through advertisement in the local newspaper. Their inclusion criteria were female sex, age between 20 and 65 years, and pain free. Exclusion criteria in both groups were any kind of anticoagulatory use, continuous anti-inflammatory drug use, opioid or steroidal use, bursitis, tendonitis, capsulitis, postoperative conditions in the neck/shoulder area, previous neck trauma, disorder of the spine, neurological disease, rheumatoid arthritis or any other systemic diseases, metabolic disease, malignancy, severe psychiatric illness, pregnancy, and difficulties understanding the Swedish language.

The number of subjects needed in order to achieve sufficient power was based on the concentration of interstitial lactate of trapezius in healthy controls and in patients with chronic trapezius myalgia reported previously^49^. Hence, using Power and Sample Size Calculation,ver. 3.0.2^50^, based on the following parameters: alpha = 0.05, power = 0.8, difference between groups = 1.7 and SD = 1.7 were found that 17 subjects in each group was needed.

Anthropometric data are presented in Table 1. The pain intensities were as expected significantly higher in CWP than in CON in the neck, shoulders, and low back (Table 1). Data concerning the included subjects with respect to psychological aspects (anxiety, depression and catastrophizing) and quality of life has essentially (i.e. not exactly equal number of subjects in the two groups in the two studies) been presented elsewhere including descriptions of the instruments used^47^. In Table 1 is reported data for the subjects participating in the present study. The CWP group had more intensive psychological symptoms even though at the group level CWP did not show definite signs of depression or anxiety according to the instrument (Hospital Anxiety and Depression Scale (HADS); cut-off values for both subscales depression and anxiety >10) used for capturing this. No significant group difference in quality of life was found (Table 1).

After receiving verbal and written information about the study, all participants signed a consent form that was in accordance with the Declaration of Helsinki. The study was granted ethical clearances by the Regional Ethical Review Board of Linköping (Dnr: M10–08, M233–09, Dnr: 2010/164–32).

### Sample handling

Biopsies were taken after a microdialysis procedure due to the fact that the participants were part of another study. In short, the participants rested in 120 min after insertion of dialysis membrane followed by a baseline period of 20 min. After this they underwent a low-force exercise during a 20 min period which was followed by a recovery period of 40 min. The membranes were then taken out and the microdialysis was ended. After this trapezius muscle biopsies were taken using Monopty^®^ BARD^®^ microbiopsy instrument (BARD Norden, Helsingborg, Sweden) from the upper trapezius muscle at the midpoint between the 7th cervical vertebra and the acromion. On extraction of the specimen, the fibers were aligned, embedded in Tissue-Tek, and frozen by immersion in isopentane, precooled with dry ice. All biopsy samples were assigned a unique identification number, thus blinding the investigator to the participant’s identity. All samples were stored at −80 °C pending analysis.

### Sample preparation

All samples were heat stabilized with Denator Stabilizer T1 (Denator, Göteborg, Sweden) and put in sample solution (9 M Urea, 4% 3-((3-cholamidopropyl) dimethylammonio)-1-propanesulfonate (CHAPS) (w/v), 65 mM dithiothreitol (DTT), 2% pharmalyte 3–10 (v/v), trace of bromophenol blue) and then homogenized by sonication 3*10 sec, incubated for 2 hours in 4 °C, followed by 1 hour centrifugation at 20,000 g. The supernatant were transferred to a new tube. 10 μL were used for measuring protein concentration by 2-D Quant Kit according the manufactures recommendation. Finally, rehydration buffer (urea 8 M, CHAPS 2%, DTT 0.3%, IPG buffer 0.5%) were added up to a final volume of 350 μL, resulting in a 100 μg protein sample, prior to isoelectric focusing (IEF).

### 2-DE analysis

The first dimension, IEF was performed on IPGphor3 (GE Healthcare), 100 μg protein was applied on IPG strips (0.5´ 3´ 180 mm), containing Immobilines giving a nonlinear pH gradient from 3 ± 10 (GE Healthcare). To assure a steady state, the focusing was performed overnight (45,000 Vhs). The strips were then equilibrated twice (or stored in −80 °C until analysis) with SDS equilibration buffer (urea 6 M, SDS 4% (w/v), glycerol 30.5% (w/v), Trizma-HCl 50 mM), first with DTT 1% (w/v), 15 minutes, secondly with iodacetamide 4.5% (w/v) with a trace of bromophenol blue, 15 minutes. The second dimension was performed vertically in an Ettan™ DALTsix Electrophoresis Unit (Amersham, Pharmacia biotech, Uppsala, Sweden), according to the manufacturers recommendations. Proteins from the IPG strip were transferred to homogenous gels cast in low fluorescent cassettes (1.0/220/270 mm, 14% T, 2.6% C) running at 80 V/10 mA* (*per gel) for about 2 hours. 2-DE then continued at up to 500 V/40 mA* (*per gel) until finished. SDS electrophoresis buffer were used (anodic, 25 mM Tris, 192 mM glycine, 0.1% (w/v) SDS and cathodic, 50 mM Tris, 384 mM glycine, 0.2% (w/v) SDS, approximate pH 8.3). Precision Plus Protein™ All Blue (Bio-Rad) was used as a molecular weight standard. Separated proteins in the analytical gels were fixed using 50% methanol/5% acetic acid/45% H_2_O solution overnight with gentle agitation. Proteins were then detected by silver staining, described elsewhere^51^, with a detection limit of about 5 ng/spot^52^. Gels from which proteins were picked for identification purposes using mass spectrometry were fixed using 10% MeOH/7% HAC solution overnight and then fluorescently stained with 500 mL SYPRO Ruby (Bio-Rad), incubated overnight. Fluorescent staining was performed according to the manufacturers staining protocol. Afterwards gels were washed and placed in deionized water. All staining and washing steps were performed with continuous gentle agitation. Proteins were visualized using a CCD camera VersaDoc™ Imaging system 4000 MP (Bio-Rad).

Finally, protein patterns in the digitized images were analyzed with PDQuest 8.0.1 (Bio-Rad) a computerized imaging 12-bit system designed for evaluations of 2-DE patterns. The amount of protein in a spot was assessed as background-corrected optical density, integrated over all pixels in the spot and expressed as integrated optical density (IOD). In order to correct for differences in total silver stain intensity between different 2-DE images, the amounts of the compared protein spots were quantified as optical density for individual spot per total protein intensity of all valid spots in the same gel. Thereby ppm-values (parts per million) for all proteins were generated that were statistically evaluated for differences between the groups.

### In-gel digestion by trypsin

Protein spots were excised using a custom made spot picker. The picked protein spots were digested with trypsin (Promega/SDS Biosciences, Falkenberg, Sweden). In short, the gel pieces were washed with a mixture of acetonitrile/ammonium bicarbonate, dehydrated with acetonitrile and incubated with 30 μL of 20 μg/mL trypsin overnight at 37 °C. The supernatant was transferred to a new tube and the peptides further extracted from the gel by incubation in 50% acetonitrile/5% trifluoroacetic acid for about 3 hours at room temperature during constant mixing. The supernatant obtained by the two steps were pooled and dried by SpeedVac.

### Protein identification by MALDI-TOF

The dried tryptic samples were dissolved in 4 μL of 0.1% trifluoroacetic acid (TFA). The peptides were mixed 1:1 with matrix solutions consisting of dihydroxybenzoic acid (DHB) (0.04 g/mL) in 70% ACN/0.3% TFA, and 1 μL was then spotted on the target plate (stainless-steel plate). Analyses of peptide masses were performed using Matrix Assisted Laser Desorption/Ionization-Time of Flight (MALDI-TOF) (Voyager-DE PRO, Applied Biosystems, Foster City, CA, USA) equipped with 337  nm N_2_ laser operated in reflector mode with delayed extraction. The mass spectra were initially calibrated externally by using a mixture of known peptides.

### Protein identification by High Resolution Mass Spectrometry

The dried tryptic samples were dissolved in 6 μL of 0.1% formic acid. The obtained peptide mixtures were analyzed by two different LC-MS/MS methods. 1) Peptides were analyzed using nano-flow HPLC system (EASY-nLC II, Thermo Fisher Scientific) or an on-line nano-flow HPLC system (EASY-nLC; Proxeon, Bruker Daltonics) in conjugation with the mass spectrometer HCTultra PTM Discovery System (Bruker Daltonics). A 100 mm × 675 mm C18 column was used for separation at a flow rate 300 nL/min. The gradient buffers were 0.1% formic acid in water (buffer A) and 0.1% formic acid in acetonitrile (buffer B) and a linear gradient from 0–100% of buffer B in 45 min was used for separation. The automated online tandem MS analysis was performed using collision induced dissociation (CID) of peptide ions. 2) Peptides were analyzed and data were acquired using Linear Trap Quadropole (LTQ) Orbitrap Velos Pro hybrid mass spectrometer (Thermo Fisher Scientific). Peptides were separated during 45 min by reverse phase chromatography on a 20 mm × 100 μm C18 pre column followed by a 100 mm × 75 μm C18 column (particle size 5 μm, NanoSeparations, Netherlands) at a flow rate 300 nL/min. A linear gradient of 0.1% formic acid in water (A) and 0.1% formic acid in acetonitrile (B) as 0–100% B in 0–45 min. Automated online analyses were performed with LTQ Orbitrap Velos Pro hybrid mass spectrometer with nano electrospray source; 240 °C capillary temperature; spray voltage, 2200 V. MS spectra were acquired in profile mode by FTMS at resolution of 30,000 (at m/z 400). Top 20 most intense multiply charged ions from were selected with an isolation window of 2.0 and fragmented in the linear ion-trap by collision induced dissociation with normalized collision energy of 30. Dynamic exclusion of sequenced peptides for 60 s and charge state filtering disqualifying singly charged peptides were activated and predictive AGC was enabled. Centroid mode was used for CID MS/MS.

### Database searches and data interpretation

The nanoLiquid Chromatography-MS/MS (nLC-MS/MS) spectra were processed by Bruker Daltonics DataAnalysis 3.4 (Bruker Daltonics, Bremen, Germany) and resulting MS/MS data were searched in NCBInr and Swiss-Prot database on MASCOT server (www.matrixscience.com). Database search parameters were set as follows: the enzyme trypsin was used; up to one missed cleavage was allowed; fixed modification included were carbamidomethylation of cysteine and oxidation of methionine; mass tolerance for MS precursor ion was 0.8 Da and for MS/MS fragment ion was 0.6 Da; and charge states were varied. Criteria for identification of a protein from the MALDI-TOF spectra were that at least 3 peptides of the protein should be identified. Using spectra from nLC-MS/MS an individual ion score >30 indicating identity or extensive homology (*p* < 0.05) and an expectation value <0.05 were used for identification. Data processing of the spectra from MALDI-TOF were performed with Data Explorer version 4.0 (Applied Biosystems). Peptide masses (plus one H^+^) of the major peaks was submitted to database search (NCBInr and SWISS-PROT) using MS-fit search engines. Parameters were set as follows: placed on species (Human), mass tolerance (50 ppm), maximum missed cleavages by trypsin ≤1; fixed modification included carbamidomethylation of cysteine and dynamic modifications were oxidation of methionine, N-terminal glutamine to pyroglutamate and N-terminus acetylation. Data from orbitrap were analyzed with MaxQuant version 1.5 to search the MS/MS spectra with trypsin specificity against the human taxonomy of the SwissProt database (release August 2014). Two missed cleavages were allowed and N-terminal acetylation and methionine oxidation were selected as variable modifications. Fixed modification was carbamidomethylation of cysteine. For MS spectra an initial mass accuracy of 6 ppm was allowed and the MS/MS tolerance was set to 0.5 Da. The FDR at the peptide spectrum matches and protein level was set to 0.01.

### Statistics

The data retrieved from the quantification, <16% was normally distributed. For comparison of group differences with univariate statistics regarding protein expression one-way ANOVA and the non-parametric Mann-Whitney *U* test were applied using IBM SPSS v.21.0 (IBM, United States) for normal distributed data and for non-normally distributed data respectively; *p* < 0.05 was considered significant. For BMI one-way ANOVA was used to test for differences between the two groups of subjects.

When investigating the multivariate correlations between the proteins and group membership Orthogonal Partial least squares discriminant analysis (OPLS-DA) was applied using SIMCA-P+ v.13.0 (UMETRICS, Umeå, Sweden)^53^. PCA was used prior to this analysis in order to check for multivariate outliers. PCA can be used to extract and display systematic variation in the data matrix. A cross validation (CV) technique was used to identify nontrivial components. Variables loading upon the same component are correlated and variables with high loadings but with different signs are negatively correlated. Significant variables with high loadings (positive or negative) are more important for the component under consideration than variables with lower absolute loadings^53^.

R^2^ describes the goodness of fit – the fraction of sum of squares of all the variables explained by a principal component^53^. Q^2^ describes the goodness of prediction – the fraction of the total variation of the variables that can be predicted by a principal component using CV methods. The purpose of applying PCA in the present study was to identify multivariate outliers using the two powerful methods available in SIMCA-P+: 1) score plots in combination with Hotelling´s T^2^ (identifies strong outliers) and 2) distance to model in X-space (identifies moderate outliers). In the present study no multivariate outliers were identified.

Variables were mean centered and scaled for unified variance (UV-scaling). The VIP variable (variable influence on projection) indicates the relevance of each X-variable pooled over all dimensions and Y-variables – the group of variables that best explain Y. VIP >1.0 combined with jack-knifed 95% confidence intervals in the regression coefficients plot not including zero were considered significant. Coefficients (PLS scaled and centered regression coefficients) were used to note the direction of the relationship (positive or negative). In the present study the analysis was made in two steps. First all proteins were included and from this analysis were selected proteins with VIP >1.0 combined with the jack-knifed confidence intervals in the coefficients plot not including zero and used in a new regression presented in the results.

Multiple linear regression (MLR) could possibly have been an alternative but it assumes that the regressor (X) variables are independent. If multi-colinearity (i.e., high correlations) occurs among the X-variables, the regression coefficients become unstable and their interpretability breaks down. MLR also assumes that a high subject-to-variables ratio is present (e.g., >5) and such requirements are not required for PLS; in fact PLS can handle subject-to-variables ratios <1. PLS in contrast to MLR can handle several Y-variables simultaneously.

## Results

A total of 216 proteins were matched on all the gels by which 211 were present in >50% of either group thus eligible for statistic validation. The apparent molecular weight and isoelectric point (p*I*) determined from 2-DE pattern were generally in agreement with the theoretical values for the identified proteins.

### Univariate group comparisons of proteins

**Figure 1:**
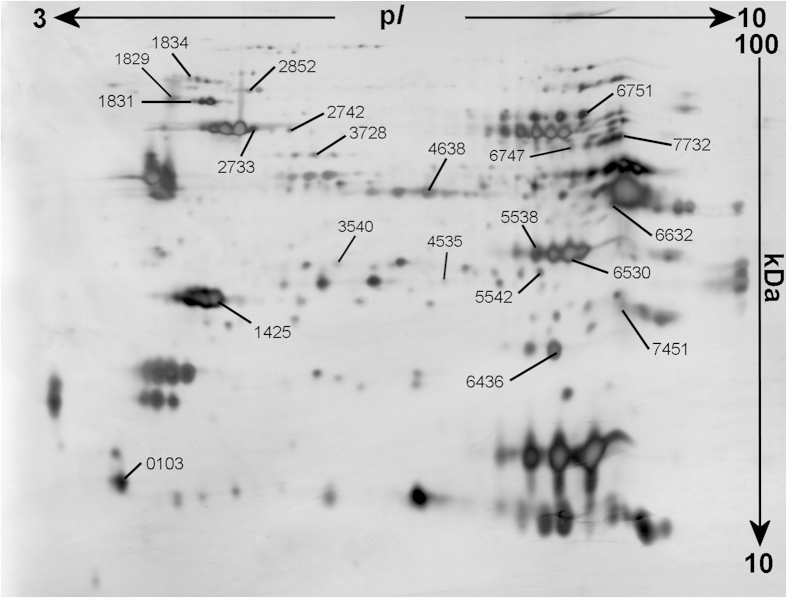
An analytical 2-DE pattern from human trapezius muscle. Numbers represent the identified proteins significantly altered according to the univariate and multivariate analysis, i.e. between groups (CWP vs CON). Horizontally the proteins are separated according to isoelectric point (p*I)* and vertically based on their molecular weight (MW).

Seventeen proteins were significantly altered between the groups (*p *< 0.05) according to the traditional statistical analyses and were selected for identification and in-gel digested for nLC/MS/MS or MALDI-TOF. Twelve of these were up-regulated (↑) in the CWP group and five were down-regulated (↓) (Fig. 1, Table 2 and Supplementary Appendix A).

The proteins were grouped based on UniProt database (http://web.expasy.org) definition on biological process in either of 4 classes: 1) stress and inflammatory (S&I, n = 3), 2) contractile (C, n = 4), 3) metabolic (M, n = 7) and 4) structural (S, n = 3) proteins. Several proteins were significantly altered in CWP compared to CON. All three stress and inflammatory proteins were up-regulated in CWP while the other types of proteins were both up- and down regulated.

### Multivariate regression analysis of group belonging

First a PCA was used to investigate whether multivariate outliers were present but this was not the case. Regression of group membership using OPLS-DA was done in two steps. First all proteins were included (R^2^ = 0.98, Q^2^ = 0.55; data not shown). Proteins with CV-VIP >1.0 were selected and then a new regression based on these selected proteins was performed (R^2^ = 0.81, Q^2^ = 0.65) with a CV-ANOVA *p* < 0.001 and thus a highly significant model explaining a high proportion of the variation in group belonging (CON or CWP) was obtained (Table 3). Also in this analysis several proteins were found to be of importance for group belonging.

Seventeen proteins were found to be significant according to our multivariate analysis of which 4 were not detected in the univariate analysis, i.e. spot number 1829, 3540, 4535 and 6747 and were identified as: protein disulfide-isomerase, heat shock protein beta-1, glutathione S-transferase Mu 2 and an isoform of fructose-bisphosphate aldolase A (Table 3). Hence, 4 proteins identified in the traditional group comparisons (Table 2) were not significant in the MVDA; i.e. spot numbers 5538 (carbonic anhydrase 3), 2733 (actin, alpha skeletal muscle), 4638 (troponin T, slow skeletal muscle) and 3728 (ankyrin repeat domain-containing protein 2).

## Discussion

Even though CWP and FMS are clinical diagnoses it is important to point out that they are syndromes. It cannot be ruled out that CWP and FMS are heterogeneous conditions based on the process of debut, activated nociceptive mechanisms or other aspects. There is no standard way to clinically identify important and robust subgroups. Chronic pain is always present in CWP/FMS but also other somatic and psychological symptoms are often present. In this study CWP/FMS patients comorbidities such as depression and anxiety were not present according to the cut-off values of the instrument used (HADS) and the perceived quality of life did not differ from CON. However, the pain per se or in combination with fear that movements will worsen pain can be associated with deconditioning and low fitness level. The present study is a cross-sectional study and alterations in proteome can thus both be due to primary nociceptive or inflammatory processes and secondary consequences e.g. deconditioning processes.

The two statistical approaches used for identification of important proteins - shown in Tables 2 and 3 - showed similarities but there were also differences. The traditional way of using univariate statistical methods to deal with a vast number of variables may often result in false positives. Traditionally, a *p*-value correction (e.g. Bonferroni) is used to correct the false discovery rate (FDR). Unfortunately, this does not only coincide with a loss of true positives it also significantly reduces the statistical power. An FDR correction can actually reduce the statistical power from 95% to 9.3% as shown previously in an mRNA microarray dataset^54^. Multivariate statistical analysis is in contrast to univariate analysis not analyzing each variable separately but instead it considers the co-variance that exists in a high degree in biological systems. Hence, we have applied MVDA in order to handle the inter-dependency between the proteins, to obtain valid and statistically un-biased results with focus upon group differences and the discussion will be focused on these proteins (Table 3). Guidelines for reporting MVDA have been suggested^55^ and were mainly applied in this study. The finally obtained model had high R^2^ and Q^2^ (0.81 and 0.65, respectively) and in order to facilitate the understanding the important proteins have been categorized in different subgroups based on UniProt database.

In the *stress and inflammatory* group, alpha-crystallin B chain and two isoforms of carbonic anhydrase 3, were up-regulated in CWP compared to CON while heat shock protein beta-1, protein disulfide-isomerase and glutathione S-transferase Mu 2 were down regulated (Table 3).

The enzyme carbonic anhydrase 3 belongs to a class of zinc metalloenzymes that catalyzes, although in a much lesser extent than carbonic anhydrase 1 and 2^56^, the reversible hydration of carbon dioxide. The protein is found in high abundance in the cytoplasm of skeletal muscle type 1 and is released in circulation upon muscle damage and possibly also reflects type I fiber abnormalities^57^. It has been suggested that carbonic anhydrase 3 plays an essential role as an antioxidative agent^58^,^59^. The protein is increased in abundance with aging^60^ while the opposite was found in hypoxia^30^. Hence, the increased levels of carbonic anhydrase 3 could indicate a higher oxidative stress in the trapezius muscle in CWP than in CON. In our recent proteomic study of microdialysate of the trapezius interstitium in the same cohort of subjects significantly up- or down- regulated isoforms of carbonic anhydrase 3 in CWP were identified, which also support the involvement of this protein in chronic myalgia^42^.

Protein disulfide-isomerase catalyzes the sulfide bonds in proteins and was down-regulated (Table 3). It also functions as a chaperone which inhibits the aggregation of other proteins^61^.

**Figure 2:**
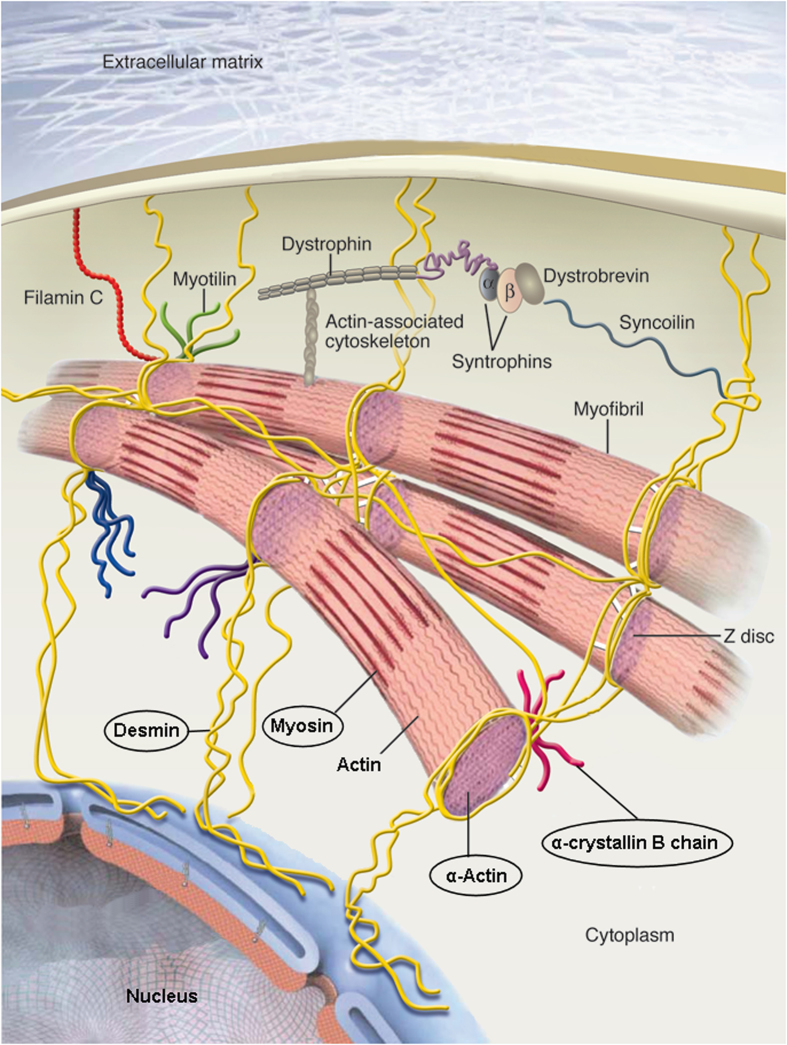
A basic structure of a myocyte. Actin and myosin are the major proteins in the contractile apparatus; the sarcomere. Desmin are predominantly found around the Z-disc linking the myofibrils together. Alpha-crystallin B chain binds to other cytoskeletal and myofibrillar proteins thus having a stabilizing effect. *Adapted with permission from The Journal of Clinical Investigation*^77^.

Heat shock protein beta-1 and alpha-crystallin B chain belongs to the small heat shock protein (HSP20) family. Alpha-crystallin B chain has chaperone-like activity, preventing aggregation of various proteins under a wide range of stress conditions^62^,^63^,^64^. The up-regulation and accumulation of the heat shock proteins including alpha-crystallin B chain may result in myofibrils more resilient to both oxidative and mechanical stresses^65^,^66^ and to protect the cytoskeleton of the myofibrils^67^ (Fig. 2). It has also been suggested that by binding to cytoskeletal and myofibrillar proteins, alpha-crystallin B chain function as stabilizers of disrupted myofibrillar structures indicating a role in muscle recovery^67^. Heat shock protein beta-1 is found in various tissues including striated muscle and is involved in stress resistance and actin organization^68^,^69^. The lowered abundance of heat shock protein beta-1 in our study may reflect an insufficient capacity or strain upon the processes of promoting muscle recovery.

Furthermore, prostaglandins are reported to play a role in induction of HSP response^70^,^71^. Arguably, our result of increased levels of alpha-crystallin B chain in the CWP group could therefore be the result of increased levels of prostaglandins which are known nociceptive/pain mediators. Glutathione S-transferase Mu 2 is involved in e.g. the detoxification of products from oxidative stress^72^. Taken together, our findings concerning S&I proteins could indicate a higher oxidative stress by altered anti-oxidative protection in CWP.

Two proteins in the *contractile* group were found to be significantly up-regulated in the CWP group, myosin light chain 1/3 skeletal muscle isoform, and myosin light chain 3 (Table 3). Myosins along with actin are the major proteins of the muscle cell and important proteins for the contraction and relaxation of muscles (Fig. 2). The myosins are motor proteins that drive movements along the actin filaments in skeletal muscle. Actins are involved in many types of cell motility, structure and are ubiquitously expressed in all eukaryotic cells. It is unclear whether the presently described alterations in contractile proteins are primarily linked to nociceptive or inflammatory mechanisms or whether they are reflecting consequences of the chronic pain condition such as deconditioning, disuse, altered activation pattern etc.^7^,^73^,^74^,^75^,^76^.

The *metabolic* group included 6 up-regulated proteins in CWP, ATP synthase subunit beta mitochondrial, triosephosphate isomerase, glyceraldehyde-3-phosphate dehydrogenase, pyruvate kinase PKM and 2 isoforms of fructose-bisphosphate aldolase A (Table 3). Two metabolic proteins, creatin kinase B-type and adenylate kinase isoenzyme 1 were down-regulated in CWP (Table 3).

Mitochondrial inner membrane ATP synthase belongs to the ATPase alpha/beta chains family and produces ATP from ADP in the respiratory system. The synthase can also work in reverse because it is dependent on the exact concentrations of the three reactants ATP, ADP, and inorganic phosphor (P_i_) in the mitochondrial matrix. If a large amount of ATP is suddenly hydrolyzed by the energy-requiring reactions in the cytosol, ATP synthase would begin to synthesize ATP to fill the gap of energy.

**Figure 3:**
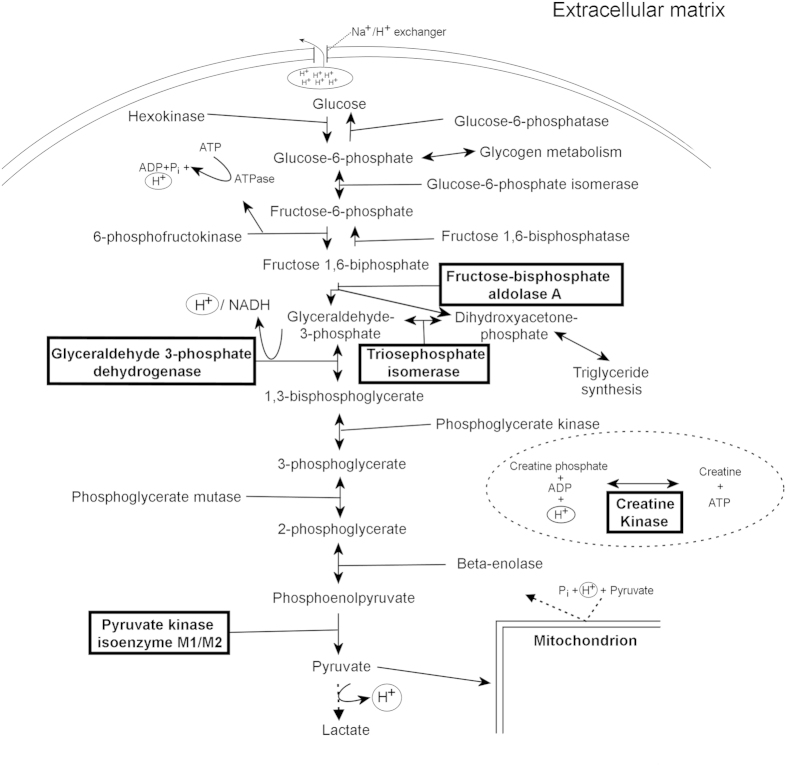
A schematic representing the metabolic pathways of the glycolysis and gluconeogenesis. The squared enzymatic proteins were all up-regulated in the CWP group compared to the healthy subjects except creatine kinase which was down-regulated. This could be explained by a higher dependence and therefore a higher strain on the glycolysis to provide energy for the skeletal muscle resulting in an accumulation of P_i_, H^+^, pyruvate and lactate.

Creatine kinase isoenzymes play a central role in energy transduction in tissues with large energy demands such as skeletal muscle. The creatine kinases catalyzes the transfer of phosphate between ATP and phosphocreatine thus buffering cellular ATP and ADP concentrations^57^ (Fig. 3). This reaction protects the muscle from entering into rigor by maintaining the ATP at the expense of phosphocreatine. Our results of decreased levels of creatine kinase B-type could result in not only lower levels of ATP, caused of the insufficient regeneration of ATP through the creatine kinase reaction, but also indirectly keeping a low muscle pH. When ADP together with P_i_ is converted into ATP a H^+^ is also consumed in the creation of creatine (Fig. 3). Considering the biochemistry, with a low cytoplasmic ATP, the muscle would end up in a “rigor-like” state i.e. a feeling of muscle stiffness which is one of the clinical symptoms of CWP^5^. Low ATP and low phosphocreatine has been detected in the quadriceps muscle of FMS and in the trapezius muscle of patients with chronic trapezius myalgia^77^,^78^.

Fructose-bisphosphate aldolase A, glyceraldehyde-3-phosphate dehydrogenase, triosephosphate isomerase and pyruvate kinase M1 are important enzymes of the glycolysis/gluconeogenesis (Fig. 3). Fructose-bisphosphate aldolase A catalyzes the breakdown of the substrate fructose 1,6-bisphosphate into glyceraldehyde 3-phosphate and dihydroxyacetone phosphate which in turn is quickly isomerized to glyceraldehyde 3-phosphate by triosephosphate isomerase. In the final step of the glycolysis, the pyruvate kinase PKM catalyzes the dephosphorylation of phosphoenolpyruvate into pyruvate. Under anaerobic conditions the muscle cells may convert the pyruvate into lactate. Increased levels of lactate in blood and increased extracellular levels of pyruvate and lactate in trapezius have recently been found in a present CWP cohort and other studies^47^,^49^,^79^. Arguably, our results could indirectly support these findings by the up-regulation of pyruvate kinase PKM. Furthermore, with multiple up-regulated metabolic proteins of which many are important enzymes in the glycolysis suggests a strain on glycolysis metabolism and could be explained by an increased need of energy to support muscle activity. Our findings could also be interpreted as muscles of patients with CWP not having enough time to metabolize or eradicate the pyruvate and/or lactate thus lowering the pH and with the potential of activating the acid-sensing ion channels (ASICs) resulting in muscle pain. These proton sensitive receptors, located at the free nerve endings have been suggested to be of importance in the induction of chronic muscle pain^80^.

The increase of glycolytic enzymes could also be a direct result of that the muscle cell must rely more heavily on an anaerobic metabolism indicating, if not a lower supply of oxygen, some obstruction or impairment of the cells capacity to use aerobic metabolism which would eventually increase P_i_, H^+^, pyruvate and lactate accumulation in the cytosol. These molecules, lactate excluded, are crucial to the mitochondrion for the production of ATP but with a higher flux through the glycolysis it exceeds what the mitochondrion is capable of processing (Fig. 3). Consequently, unbuffered protons leave the cell via the Na^+^/H^+^ exchangers and lactate^−^/H^+^ symporters entering the blood stream thus altering the blood pH. Because the tricarboxylic acid cycle in the mitochondria demands oxygen the pyruvate cannot be oxidized thus theoretically contributing to an increase of cytoplasmic pyruvate and lactate which subsequently through proton release would activate ASICs. Whether a decrease in pH is caused by the increase of lactate is still debated^81^,^82^,^83^. Traditionally it has been explained by proton release through the formation of lactate from pyruvate. Another explanation is hydrolysis of ATP generated from the glycolysis as the major source to the decrease of pH (Fig. 3). Notably, additional H^+^ accumulation could be produced from the glyceraldehyde-3-phosphate dehydrogenase reaction^82^; the enzyme responsible was up-regulated in the CWP group (Fig. 3). Low pH also activates the transient receptor potential receptor subtype 1 (TRPV1) which is also sensitive to heat and becomes even more so when pH drops. A proteomic study^41^ of local trapezius myalgia also identified quantitative alterations of the metabolic proteins fructose-bisphosphate aldolase and pyruvate kinase PKM although in contrast to our results the proteins were down-regulated which can be the result from the fact that it is two different pain conditions and also methodological differences.

Interestingly, similar results of up-regulated metabolic enzymes have been found in a cold- and overfeeding induced proteomic study of the skeletal muscle^35^. After overfeeding with a diet of 160% energy balance an increase in fructose-bisphosphate aldolase A, triosephosphate isomerase, glyceraldehyde-3-phosphate dehydrogenase and a decrease in creatine kinase M chain could be observed. CWP had a significantly higher BMI than CON (*p* < 0.05) which could indicate different food-intake between the groups.

In conclusion with respect to the metabolic proteins the results could indicate a strain on the metabolic glycolytic pathways in CWP.

The *structural* proteins keratin, type II cytoskeletal 1 was up-regulated while desmin was down-regulated in CWP. Both these proteins were also identified as important in the muscle biopsy study of work related chronic trapezius myalgia^41^. Desmin and keratin, type II belong to the intermediate filament (IF) family. These types of proteins are components of the cytoskeleton and nuclear envelope and form structures between microtubules and microfilaments and are involved in numerous processes in the organization of the cell (Fig. 2). Keratin, type II is believed to regulate protein kinase C via binding to integrin beta-1^84^. Desmin are also responsible for mitochondrial shape, function and positioning to regions of high energy demand in the skeletal muscle cell^85^,^86^. Interestingly there are reports of increased prevalence of muscle fibers with disturbed distribution of mitochondria (e.g. ragged-red fibers) in the trapezius of patients with CWP (i.e. FMS)^21^,^87^. IFs have previously been reported to provide flexible intracellular scaffolding to resist oxidative stress utilized upon cells^88^,^89^. Mutations of desmin appear to be related to muscle weakness as in limb girdle dystrophy^90^ and in desmin myopathy^91^,^92^. In the latter condition the mutations affect amino acids that are critical for the filament structure^93^,^94^. To summarize, the alterations of these structural proteins in CWP may indicate a reduced ability of regeneration of the cytoskeleton and eventually disturbances in function and distribution of mitochondria.

Although, 2-DE is a useful comprehensive method it is also important to keep in mind that this method only detect the soluble and to some extent only the most abundant proteins and that what can be visualized by the staining method used in this study is still only a fraction of the proteins expressed in human trapezius muscle, and it can be expected that there are still proteins not detectable by 2-DE that have not been evaluated. Also, the present results with significant protein alterations in CWP must be confirmed in other cohorts. In such future studies of CWP or FMS it is important to also control for other symptoms and comorbidities, fear-avoidance and fitness level. Prospective proteomic studies - e.g., including evaluations of exercise interventions - are necessary in order to understand which proteomic alterations are linked to primary nociceptive processes and pain and which are related to secondary consequences such as deconditioning.

Using a proteomic explorative approach in combination with multivariate modeling we have identified 17 proteins (4 different classes) that clearly and significantly separated CWP from CON (explained variation 81%). The proteins consist of enzymes involved in metabolic pathways, proteins associated with muscle damage, muscle recovery and oxidative stress. Although single proteins has been suggested as biomarkers for chronic muscle pain, this and other recent proteomic studies indicate the existence of comprehensive alterations and therefore protein clusters or *bioclusters* are far more realistic. The described alterations - e.g. for stress and inflammatory proteins - in aching muscles of CWP may give support to a suggestion that peripheral factors are of importance in maintaining CWP. However, it cannot be ruled out that the changes observed are secondary consequences of nociceptive and pain processes in the central nervous system. In forthcoming studies the relationships between the muscle proteome and pain intensity and pain sensitivity will be explored.

## Additional Information

**How to cite this article**: Olausson, P. *et al.* Protein alterations in women with chronic widespread pain – An explorative proteomic study of the trapezius muscle. *Sci. Rep.* **5**, 11894; doi: 10.1038/srep11894 (2015).

## Supplementary Material

Supplementary Appendix A

## Figures and Tables

**Table 1:** Age and anthropometric data together with pain intensity.

Variables	CON (n = 19)	CWP (n = 18)	Statistics (p-value)
Age (years)	41.2 ± 10.6	48.6 ± 9.7	0.035*
Height (cm)	168.7 ± 7.7	167.6 ± 5.1	0.613
Weight (kg)	68.5 ± 12.8	76.8 ± 17.3	0.110
Body Mass Index (kg/m^2^)	23.9 ± 3.1	27.2 ± 5.5	0.034*
Pain intensity – neck (NRS)	0.1 ± 0.5	5.5 ± 2.1	<0.001*
Pain intensity – shoulders (NRS)	0.0 ± 0.0	5.7 ± 1.7	<0.001*
Pain intensity – low back (NRS)	0.1 ± 0.5	5.9 ± 1.5	<0.001*
HADS-Anxiety	2.5 ± 2.3	6.9 ± 3.8	<0.001
HADS-Depression	1.3 ± 1.7	5.6 ± 3.4	<0.001
PCS	7.5 ± 6.8	10.6 ± 5.3	0.154
QOL-S	92.4 ± 11.2	86.6 ± 11.2	0.147
Nos. with FMS (n)	0	15	NA

Other symptoms (depressive and anxiety), catastrophizing, and quality of life gathered from a questionnaire completed by the healthy controls (CON) and the patients with chronic widespread pain (CWP); mean ± one standard deviation (SD) is given. Numbers of subjects with fibromyalgia syndrome is also reported. Furthest to the right is the result of the statistical comparison between the two groups (*p*-value).

NRS = numeric rating scale; HADS-Anxiety = Hospital Anxiety and Depression Scale - subscale anxiety; HADS-Depression = Hospital Anxiety and Depression Scale - subscale depression; PCS = Pain Catastrophizing Scale- total score; QOL-S = Quality of Life Scale; FMS = Fibromyalgia syndrome; NA = not applicable.

^*^denotes significant difference.

**Table 2:** Significantly (*p* < 0.05) altered proteins identified from the trapezius muscle by nLC-MS/MS and MALDI-TOF.

Spot no.	Protein	Ratio CWP vs CON	Type
5538 **ƪ**	Carbonic anhydrase 3	↑	S&I
6436	Alpha-crystallin B chain	↑	S&I
6530	Carbonic anhydrase 3	↑	S&I
0103	Myosin light chain 1/3, skeletal muscle isoform	↑	C
1425	Myosin light chain 3	↑	C
2733 **ƪ**	Actin, alpha skeletal muscle	↑	C
4638 **ƪ**	Troponin T, slow skeletal muscle	↓	C
1831	ATP synthase subunit beta, mitochondrial	↑	M
2742	Creatine Kinase B-type	↓	M
5542	Triosephosphate isomerase	↑	M
6632	Glyceraldehyde-3-phosphate dehydrogenase	↑	M
6751	Pyruvate kinase PKM	↑	M
7451	Adenylate kinase isoenzyme 1	↓	M
7732	Fructose-bisphosphate aldolase A	↑	M
1834	Keratin, type II cytoskeletal 1	↑	S
2852	Desmin	↓	S
3728 **ƪ**	Ankyrin repeat domain-containing protein 2	↓	S

↑ = Up-regulated; ↓ = Down-regulated in patients with CWP compared to the CON. The proteins were divided based on UniProt database (http://web.expasy.org) definition on biological process in different groups (labelled Type); S&I = stress and inflammatory, C = contractile, M = metabolic and S = structural proteins. Proteins marked with ƪ were not significant in the OPLS-DA (Table 3).

**Table 3:** OPLS-DA regression of group membership (CWP vs. CON).

Spot no.	Protein	VIP	CoeffCS	Type
1831	ATP synthase subunit beta, mitochondrial	1.66	+	M
5542	Triosephosphate isomerase	1.65	+	M
2742	Creatine Kinase B-type	1.45	−	M
1829*	Protein disulfide-isomerase	1.27	−	S&I
7732	Fructose-bisphosphate aldolase A	1.21	+	M
1834	Keratin, type II cytoskeletal 1	1.20	+	S
6747*	Fructose-bisphosphate aldolase A	1.18	+	M
7451	Adenylate kinase isoenzyme 1	1.17	−	M
0103	Myosin light chain 1/3, skeletal muscle isoform	1.13	+	C
6436	Alpha-crystallin B chain	1.11	+	S&I
6751	Pyruvate kinase PKM	1.10	+	M
6530	Carbonic anhydrase 3	1.09	+	S&I
2852	Desmin	1.08	−	S
6632	Glyceraldehyde-3-phosphate dehydrogenase	1.05	+	M
4535*	Glutathione S-transferase Mu 2	1.02	−	S&I
1425	Myosin light chain 3	1.01	+	C
3540*	Heat shock protein beta-1	1.00	−	S&I

For each protein is reported CV VIP (VIP >1.0 is significant and only proteins with VIP >1 are shown) and signs of the coefficient (+ or −) (R^2^ = 0.81, Q2 = 0.65). Proteins with positive coefficients are higher in CWP than in CON and vice versa for negative coefficients. The proteins were divided based on UniProt database (http://web.expasy.org) definition on biological process in different groups (labelled Type); M = metabolic, S&I = stress and inflammatory, S = structural and C = contractile proteins. Proteins marked with *were not significant in the univariate group comparison (Table 2).
