# Supplementary material for: Protein alterations in women with chronic widespread pain – An explorative proteomic study of the trapezius muscle
Source: Sci Rep. 2015 Jul 7;5:11894. doi: 10.1038/srep11894 (PMC4493691; doi:10.1038/srep11894)
Supplement: Supplementary Appendix A [file srep11894-s1.pdf]

Protein alterations in women with chronic widespread pain – An explorative proteomic study of the trapezius muscle

Patrik Olausson[1], Björn Gerdle[1], Nazdar Ghafouri[1], Dick Sjöström[1], Emelie Blixt[1], and Bijar Ghafouri[1]

Supplementary Appendix A

Spot no.	Protein	Accession no.	MW(Da)/p*I*	Matched peptides or Unique Peptides*	Mascot Score/ Mowse score(*)/ Orbitrap (-)	Sequence coverage (%)	Ratio CWP vs CON
**Stress and inflammatory**							
1829	Protein disulfide-isomerase	P07237	57116/4.8	25*	-	53	↓
3540	Heat shock protein beta-1	P04792	22783/6.0	5	3.03e+6	28	↓
4535	Glutathione S-transferase Mu 2	P28161	25745/6.0	13*	-	63	↓
5538	Carbonic anhydrase 3	P07451	29824/6.9	6	175	25	↑
6436	Alpha-crystallin B chain	P02511	20146/6.8	5	137	37	↑
6530	Carbonic anhydrase 3	P07451	29824/6.9	13	390	34	↑
**Contractile**							
0103	Myosin light chain 1/3, skeletal muscle isoform	P05976	21189/5.0	4	157	18	↑

1425	Myosin light chain 3	P08590	22089/5.0	17	530	65	↑
2733	Actin, alpha skeletal muscle	P68133	42051/5.2	17	7.50e+7*	50	↑
4638	Troponin T, slow skeletal muscle	P13805	32948/5.9	13	2.8e+07*	38	↓
**Metabolic**							
1831	ATP synthase subunit beta, mitochondrial	P06576	56525/5.3	8	292	16	↑
2742	Creatine Kinase B-type	P12277	42645/5.3	14	4.24e+6*	49	↓
5542	Triosephosphate isomerase	P60174	30791/5.7	10	61	52	↑
6632	Glyceraldehyde-3-phosphate dehydrogenase	P04406	36201/8.6	4	156	25	↑
6747	Fructose-bisphosphate aldolase A	P04075	39851/8.3	11	1.06e+4	25	↑
6751	Pyruvate kinase PKM	P14618	58470/8.0	11	301	24	↑
7451	Adenylate kinase isoenzyme 1	P00568	21635/8.7	9	77	55	↓

7732	Fructose-bisphosphate aldolase A	P04075	39851/8.3	13	339	35	↑
**Structural**							
1834	Keratin, type II cytoskeletal 1	P04264	66170/8.2	9	365	16	↑
2852	Desmin	P17661	53536/5.2	38	3.21e+16*	78	↓
3728	Ankyrin repeat domain-containing protein 2	Q9GZV1	40006/5.7	5	240	27	↓
